# The food contaminant deoxynivalenol provokes metabolic impairments resulting in non-alcoholic fatty liver (NAFL) in mice

**DOI:** 10.1038/s41598-020-68712-w

**Published:** 2020-07-21

**Authors:** Rym Barbouche, Stéphanie Gaigé, Coraline Airault, Kevin Poirot, Michel Dallaporta, Jean-Denis Troadec, Anne Abysique

**Affiliations:** Laboratoire de Neurosciences Cognitives, UMR CNRS 7291, Université Aix-Marseille, Campus St Charles, 3 Place Victor Hugo, 13331 Marseille Cedex 3, France

**Keywords:** Metabolism, Environmental impact, Physiology, Gastroenterology

## Abstract

The ribotoxin deoxynivalenol (DON) is a trichothecene found on cereals responsible for mycotoxicosis in both humans and farm animals. DON toxicity is characterized by reduced food intake, diminished nutritional efficiency and immunologic effects. The present study was designed to further characterize the alterations in energy metabolism induced by DON intoxication. We demonstrated that acute DON intoxication triggered liver steatosis associated with an altered expression of genes related to lipids oxidation, lipogenesis and lipolysis. This steatosis was concomitant to anorexia, hypoglycemia and a paradoxical transient insulin release. DON treatment resulted also in stimulation of central autonomic network regulating sympathetic outflow and adrenaline and glucocorticoids secretion. Furthermore, an increased expression of genes linked to inflammation and reticulum endoplasmic stress was observed in the liver of DON-treated mice. Finally, we propose that lipids mobilization from adipose tissues (AT) induced by DON intoxication drives hepatic steatosis since (1) genes encoding lipolytic enzymes were up-regulated in AT and (2) plasma concentration of triglycerides (TGs) and non-esterified fatty acids were increased during DON intoxication. Altogether, these data demonstrate that DON induced hormonal and metabolic dysregulations associated with a spectrum of hepatic abnormalities, evocative of a non-alcoholic fatty liver disease.

## Introduction

Trichothecenes are toxins produced by fungi and represent a worldwide threat for agricultural production, food industries and both animal and human health. A growing number of countries introduce regulations or guidelines for food and feed contamination levels of the most prevalent trichothecene, deoxynivalenol (DON), on the basis of its ability to cause growth suppression^1^. With the improvement of analytical tools, evaluation of food contamination and human exposure revealed that a proportion of worldwide population is chronically exposed to DON doses close or exceeding the provisional maximum tolerable daily dose set by the joint FAO/WHO Expert Committee on Food Additives at 1 µg/kg body weight^2^. In this context, children appear as a population with a high risk of dietary exposure to this mycotoxin, due to their long-term consumption of wheat flour and corn-based products, which are often heavily contaminated^3–7^. The European Commission has established regulations for the maximum level of DON in crude cereals, and in processed cereal-based baby foods as 1,250 and 200 µg/kg, respectively (Commission Regulation No. 1881/2006). Accordingly, a better understanding of DON impact on health is needed. Exposition to low or moderate DON doses induces anorexia, vomiting and reduced weight gain. We and others having contributed to address the mechanisms by which DON induce these symptoms and revealed a multifaceted action targeting different organs and causing dysregulation in neuroendocrine, nervous and immune responses^1,8^. Acute intraperitoneal (i.p.) exposure to DON was reported to increase rapidly plasma levels of cholecystokinin and of the satiety hormones peptide YY which contributed to DON-induced anorexia^9^. Moreover, we previously shown that DON triggers the activation of specific neuronal populations dedicated to food intake control and energy metabolism at the hypothalamus and the brainstem levels upon acute oral gavage^10^. A hallmark of DON toxicosis at moderate doses is the activation of the innate immune system and the resulting surge in the expression of pro-inflammatory cytokines^11^. Using acute DON oral exposure in mice as a model, several studies have shown up-regulation of pro-inflammatory cytokines in various tissues i.e. spleen, liver, kidney, small intestine and brain^12,13^.

The liver constitutes the main detoxifying organ of the body and participates in detoxification of ingested xenobiotics. Detoxification of DON, which involves the formation of DON-glucuronide metabolites (DON-GlcA) less toxic than the parental toxin, certainly starts in gut level but the liver may also contribute. Liver microsomes extracted from animals and humans have been shown to be able to transform DON in glucuronide-DON (see for review^14^). Besides its detoxifying function, the liver plays a central role in body energy metabolism. The liver is a major site for synthesis, metabolism, storage and redistribution of carbohydrates, proteins and lipids. Moreover, it acts as a hub to metabolically connect various tissues, including skeletal muscle and adipose tissue. Liver energy metabolism is tightly controlled by multiple nutrients, hormonal, and neuronal signals that regulate glucose, lipid, and amino acid anabolism and catabolism. Moreover, in the liver, endoplasmic reticulum (ER) stress leads to lipid accumulation, accompanied by modification of metabolic gene expression^15^. So, dysfunction of liver metabolism causes or predisposes to non-alcoholic fatty liver disease (NAFLD) and/or type 2 diabetes. Results from previous studies have reported that DON can induce hepatotoxicity associated with oxidative stress and apoptosis, but some others illustrated opposite results (see^16^ for review). In this context, the present work was designed to determine how the liver metabolism is affected during DON intoxication and how far it contributes to the global alterations in energy metabolism induced by this mycotoxin.

## Materials and methods

### Animal housing

Experiments were performed on adult male C57BL/6 mice of 20–25 g body weight (bw) (Charles River, L’Arbresle, France). All animals were individually housed in a pathogen-free facility at controlled temperature on a 12/12 h light/dark cycle (lights on at 7 h a.m.) with standard powder diet (AO4 P2.5, SAFE UAR) and water available ad libitum. All experiments were performed at 21 °C.

### Ethics statement

Experiments carried out in this study were performed in strict accordance with European Economic Community guidelines (86/609/EEC). All protocols were approved by the local committees’ recommendations (C-13-055-6, Aix-Marseille University) for the care and use of laboratory animals.

### *Per os* administration of DON

DON treatment was performed as previously described^10^. Briefly, after one week of habituation to oral administration procedure using a 22-gauge intubation needle (Popper and Sons), mice were administered orally by 12.5 mg/kg bw DON (D-0156, Sigma Chemical Co.) dissolved in H_2_O or H_2_O alone. For food intake measurement, DON was administered orally one hour prior to the beginning of the dark phase.

### Glycaemia measurements

Mice were fasted 3 h before DON administration. DON (12.5 mg/kg bw) was administrated by gavage 30 min before lights off. Glycaemia measurements were carried out during the dark phase. Chow was not reintroduced during the glycaemia measurements to exclude any impact of a differential food intake between the two experimental groups. Blood samples were collected in heparin (25,000 UI/5 mL) from retro-orbital punctures and glucose blood was directly applied to a strip to allow glucose detection with a glucose meter (Accu-Chek Adv., Roche Diagnostics Corp.).

### Tissues collection

Mice were anaesthetized using i.p. injection of ketamine (120 mg/kg; Ketamine 1,000 Virbac) and xylazine (16 mg/kg; Rompun, Bayer Santé, France) and sacrificed by decapitation at different time after *per os* vehicle or DON administration. To avoid any impact of a differential food intake between the two experimental groups, chow was withdrawn after treatment. Both the liver, GAT and RPAT tissues were collected, rinsed in phosphate buffer saline (PBS), snap frozen in liquid nitrogen and stored at – 80 °C until further analysis. For histological studies, liver was minced into small pieces (~ 1 mm^3^) before fixation by immersion in paraformaldehyde (PFA) 4% overnight. After rinsing in PBS liver samples were cryoprotected for 24 h in 30% sucrose at 4 °C and then frozen in isopentane (− 40 °C).

### Analysis of plasma samples

Blood samples were centrifuged (3,000*g*, 15 min, 4 °C). Subsequently, plasmas were collected and quickly frozen at − 80 °C until use. Mouse enzyme-linked immunosorbent assay (ELISA) kits were used to measure plasma concentrations of insulin (Crystal Chem, USA), corticosterone (Crystal Chem, USA) and epinephrine (Mybiosource, USA) according to the manufacturer’s instructions. Quantitative determination of triglycerides (TGs) and non-esterified fatty acids (NEFAs) concentrations were assessed by enzymatic colorimetric method assay kits (Bioassay Systems, USA and FUGIFILM Wako Chemicals Europe, respectively) according to the manufacturer’s instructions.

### Western Blot

Liver pieces were homogenized in RIPA buffer (50 mM Tris pH 8.0, 0.1% sodium dodecyl sulfate, 1% Triton X-100, 0.5% sodium deoxycholate, 150 mM NaCl, and 1 mM EDTA) supplemented with protease inhibitors cocktail (bimake.com, UK) and maintained under constant agitation at 4 °C for 1 h. Then extracts were centrifuged at 12,000*g* for 20 min at 4 °C to remove tissue debris. The supernatants were collected for assessment of protein concentration by Bicinchoninic Acid (BCA) Protein Assay method (Novagen, Germany). Equivalent amounts of protein (30 μg) were separated on 12% SDS-PAGE gel and transferred onto nitrocellulose membranes (Amersham, Germany) to form blots. The transfer efficiency is checked by Ponceau S coloring (Sigma). Blots were blocked for 30 min with 2% casein in PBS- 0, 1% Tween 20 and incubated 1.5 h at room temperature with rabbit monoclonal anti-ADRP antibody [EPR3713] (Abcam) at 1:500 dilution. Blots were then incubated for 1 h at room temperature with anti-rabbit IgG heavy and light chain antibody (1:10,000; Bethyl Laboratories) conjugated to horseradish peroxidase (HRP). For loading control, blots were further stripped and re-probed with glyceraldehyde-3-phosphate dehydrogenase (GAPDH) mouse monoclonal antibody (1:5,000; Proteintech, USA) followed by HRP-conjugated goat anti-mouse secondary antibody (1:2,500, Sigma Aldrich). Bands were visualized using the colorimetric system TMB one component HRP membrane substrate (SurModics, USA) and quantified by densitometry using Image J software (NIH, USA).

### Quantitative PCR

Total RNA was extracted from frozen organ using TRI Reagent (Sigma–Aldrich) according to the manufacturer’s instructions. Samples were treated with AccuRT Genomic DNA Removal Kit, (G488, ABM, Euromedex). Reverse transcription was realized using OneScript cDNA Synthesis Kit (G233, ABM Euromedex) in the presence of random hexamer primers (Promega). Gene expression analysis by real time PCR was performed using the LightCycler 480 System (Roche Applied Science). The equivalent of 10 or 40 ng initial RNA, for adipose tissues and liver respectively, was subjected to PCR amplification (Applied Biosystems). The generation of specific PCR products was confirmed by melting-curve analysis (see Table 1). The stability of housekeeping genes used for data normalization is critical and previous work has reported that NAFLD could affect their expression^17^. Accordingly, four different reference genes i.e. GAPDH, RNA Polymerase II Subunit A (POLR2a), U6 and Tyrosine 3-Monooxygenase/Tryptophan 5-Monooxygenase Activation Protein Zeta (YWASZ) were used in the present study. U6 and GAPDH were found to be stable and thus used as internal reference gene.

**Table 1:** Primers sequences used for SYBR Green assays.

Gene product	RefSeq	Primers	
		Fw	Rv
Acaca	NM_133360.2	GGGAACATCCCCACGCTAAA	GAAAGAGACCATTCCGCCCA
Acadm	NM_007382.5	CACAACACTCGAAAGCGGC	GATGAGAGGGAACGGGTACTC
Acox1	NM_015729.3	TCACAGCAGTGGGATTCCAAA	CGGCAGGTCATTCAAGTACG
Atf6	NM_001081304.1	ACGAGGTGGTGTCAGAGAAC	GCAGGGCTCACACTAGGTTT
Cact	NM_020520.5	CAGATTCAGGCTTCTTCAGGG	CCACTGGCAGGAACATCTC
CHOP	NM_007837.4	CCCCAGGAAACGAAGAGGAA	GACTGGAATCTGGAGAGCGA
Cpt1	NM_153679.2	TGCTGTCTTCACTGAGTTCC	CACCAGCAAGAAAGTCATTCCA
Cpt2	NM_009949.2	ACAGCATCGTACCCACCAT	ACACAACACTTCTGTCTTCCTGA
Cyp4a10	NM_010011.3	TCCCAAGTGCCTTTCCTAGA	GGGGTTAGCATCCTCCTGT
Fasn	NM_007988.3	GCCTCACTGCCATCCAGATT	CCCAAGGAGTGCCCAATGAT
Hmgcr	NM_008255.2	GTCGCTGGTCCTAGAGCTT	CCTTTGGGTTACGGGGTTTG
IL-1β	NM_008361.3	GACCCCAAAAGATGAAGGGCT	AAGGTCCACGGGAAAGACAC
IL-6	NM_031168.2	ACAACCACGGCCTTCCCTAC	ATCAGAATTGCCATTGCACAAC
Ire1	NM_023913.2	ACCTGCCCAAACATCGAGAA	CCTCCACATCCTGAGATACGG
HSL	NM_010719.5	CCTGGAACTAAGTGGACGCA	ACATCTCGGGGCTGTCTGAA
MGL	NM_001166251.1	CTCCACAGAATGTTCCCTACCA	TGTCCAGCCCCTTCAACATA
PEPCK	NM_1044.3	ACTGACAGACTCGCCCTATGTG	GTTGCAGGCCCAGTTGTTGA
Perk1	NM_010121.3	AGAACAGCGTGTACTTAGGGA	AGACCCAACCAAGACAGGA
ATGL	NM_025802.3	TCCAACATGCTACCAGTGCG	AGCCACTCCAACAAGCGG
Scd1	NM_009127.4	CAAGCTGGAGTACGTCTGG	GCGCTGGTCATGTAGTAGAA
Srebf1	NM_011480.4	ACTCCCTCTGATGCTACGG	TGGGTCCAATTAGAGCCATCTC
Srebf2	NM_033218.1	GCGACCAGCTTTCAAGTCC	CATTGGCTGTCTGCGTCAAC
TNFα	NM_013693.3	TCTTCTCATTCCTGCTTGTGG	GAGGCCATTTGGGAACTTCT
XBP1	NM_001271730.1	CTGGCGTAGACGTTTCCTGG	GCCTTACTCCACTCCCCTTGG
GAPDH	NM_001289726.1	TTCTCAAGCTCATTTCCTGGTATG	GGATAGGGCCTCTCTTGCTCA
POLR2a	NM_001291068.1	GCACCATCAAGAGAGTGCAG	GACCTCCCTCCGTTGTTTC
U6	NR_003027.2	GCTTCGGCAGCACATATACTAAAAT	CGCTTCACGAATTTGCGTGTCAT
YWASZ	NM_001253805.1	ACATCTGCAACGATGTACTGTCT	TGCTGTGACTGGTCCACAAT

### Tissue histology using Oil Red O and Periodic Acid-Schiff staining

Thick sections (10 µm) were prepared from fixed liver samples with a cryostat (Leica CM3050, France). Some sections were staining with Oil Red O (ORO) to identify lipid inclusions in hepatocytes and for alternative serial sections Periodic Acid-Schiff (PAS) staining was performed to visualize hepatic glycogen particles. ORO working solution was prepared as described by^18^. ORO staining protocol of hepatic sections was adapted from^19^. Hepatic sections were immersed in propylene glycol (100%) for 2 min, then covered with ORO working solution for 6 min, immersed in propylene glycol (85%) for 1 min and washed two times (5 min) in H_2_O. For microscopic observations, sections were mounted in Mowiol solution. For PAS staining (adapted from Sigma-Aldrich’s instructions), sections were incubated in periodic acid for 5 min and washed three times (3 min) in H_2_O. Then sections were incubated in Schiff’s solution for 5 min and washed as described above. Finally, sections were dehydrated in a graded series of ethanol and in xylene solution, and then mounted in Eukitt solution for microscopic observations. Microphotographs (×20) were acquired using a Nikon Eclipse E600 light microscope coupled to a DXM 1,200 Camera and ACT-1 software. Using the NIH Image J software, for each condition, the number of lipid inclusions per surface unity (0.1 mm^2^) was counted and the glycogen area per surface unity (0.1 mm^2^) was quantified.

### c-Fos immunohistochemistry

The c-Fos immunochemistry was carried out as described previously^20^ except for the secondary antibody, the incubation time of which was 1.5 h instead of 2 h. For each animal, c-Fos immunostaining analysis was performed by counting the positive nuclei on four non-consecutive hemisections using microphotographs acquired by a tenfold lens with a DMX 1,200 camera (Nikon) coupled to ACT-1 software. The microscope was set at a specific illumination level, as was the camera exposure time. c-Fos positive nuclei were then counted on these pictures by computer-assisted morphometry using the ImageJ software as previously described^10^.

### Statistical analysis

All results are presented as mean ± SEM. Statistical analyses were performed using Graphpad Prism 6.05. Comparison between two groups was performed using unpaired 2-tailed Student's *t* test. Pearson correlation analysis was used to quantify relationships between variables of interest. Two ways ANOVAs followed by Bonferroni’s multiple comparisons were used to assess DON’s effects at different time post treatment. *P* values less than 0.05 were considered significant.

## Results

### Acute DON intoxication induces lipid accumulation into liver

**Figure 1:**
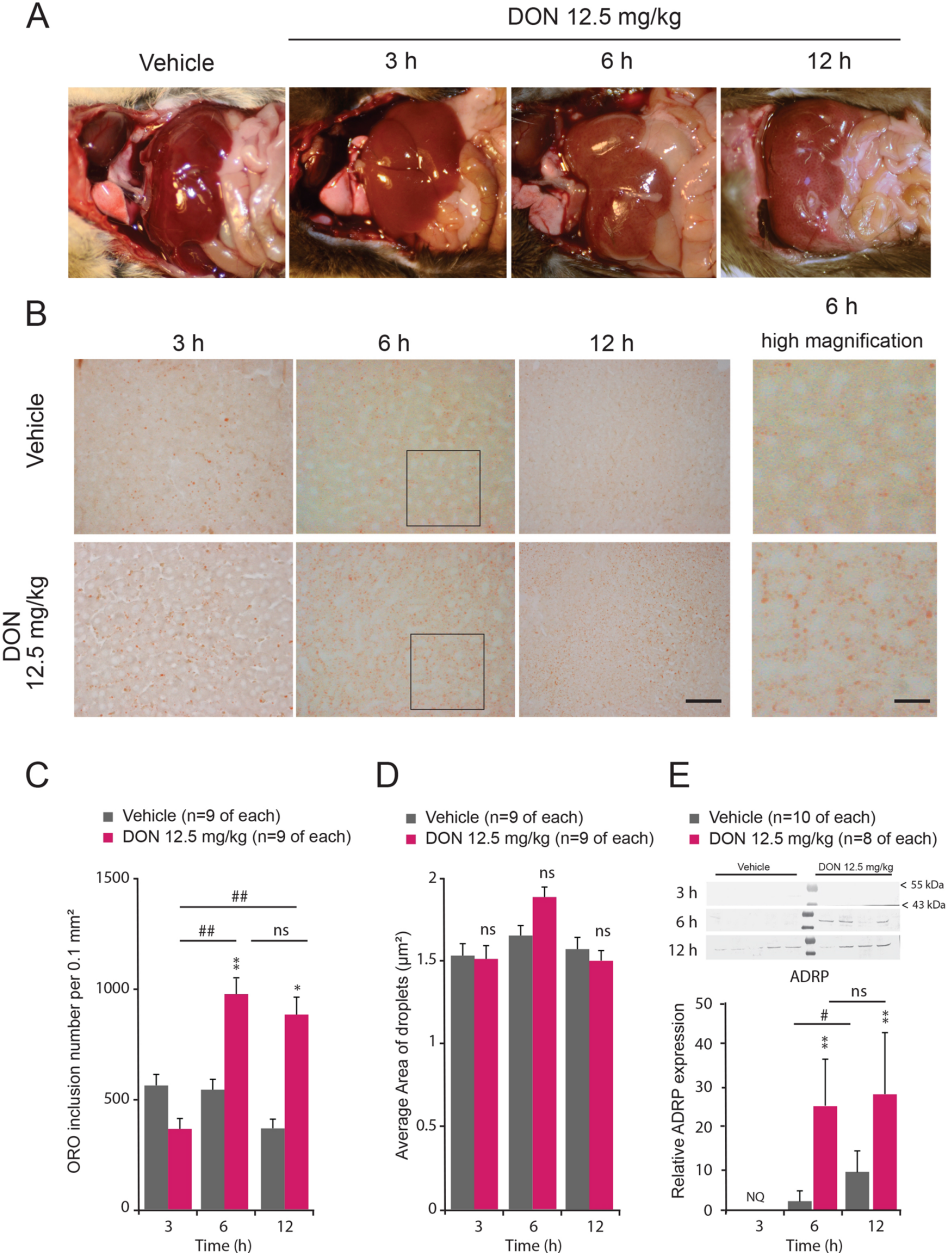
(**A**) In situ liver photographs from control and DON-treated animals. (**B**) Representative microphotographs of frozen liver section stained with ORO observed in vehicle (distilled water) or 12.5 mg/kg DON for the indicated times. High magnifications of ORO staining observed 6 h after vehicle or 12.5 mg/kg administration. Areas where these high magnifications originate are visualized by square in 6 h photographs. Scale bars = 100 and 30 µm in low and high magnification respectively. (**C**, **D**) Quantification of ORO positive inclusion number per surface unit (**C**) and size (**D**) in liver of vehicle and DON-treated mice. (**E**) Representative Western blot and quantitative comparison of ADRP protein expression (48 kDa) assessed in vehicle and DON-treated mice. GAPDH expression was used to normalize the results (not shown). NQ indicates non quantifiable data. A two-way analysis of variance test was performed between time series (3, 6 and 12 h) of DON-treated (12.5 mg/kg bw) and untreated mice (panels **C**, **D** and **E**). *P < 0.05 and **P < 0.01 significantly different from control mice. ^#^*P* < 0.05 and ^##^*P* < 0.01 significant difference between time point of DON-treated mice. ns: no significant difference.

DON was acutely administered *per os* at a dose of 12.5 mg/kg bw throughout this study. As shown in Fig. 1A, livers of DON-treated mice, especially at 6 h post-treatment, appeared slightly yellow suggesting increased lipids storage. We then confirmed lipid liver accumulation by ORO staining, which detects neutral lipids and revealed very little accumulation in vehicle-treated animals, but substantial steatosis in the liver of DON-treated animals (Fig. 1B). At 3 h post DON ingestion, ORO coloration did not reveal a significant increase in lipid droplets number although some animals displayed a strong coloration. Hepatic lipid content increased more substantially at 6 h DON post-treatment (Fig. 1B). At this time point, the number of lipid droplets was increased by ~ 70% in the DON-treated group compared to the control (Fig. 1C) while their size remained unchanged (Fig. 1D). Twelve hours after DON treatment, the number of lipid droplets remained significantly higher than in control condition (Fig. 1C). Moreover, ADRP protein expression, that has been ascribed a role in cellular fatty acid uptake and storage, was also strongly up-regulated in the DON-treated group at 6 and 12 h after challenge (Fig. 1E). Note that ADRP expression slightly increased in control group over the time (6 and 12 h), this could reflect a small steatosis induced by fasting^21^ since chow was withdrawn after treatment to avoid any impact of a differential food intake between the two experimental groups.

### DON intoxication results in metabolic and hormonal alterations

**Figure 2:**
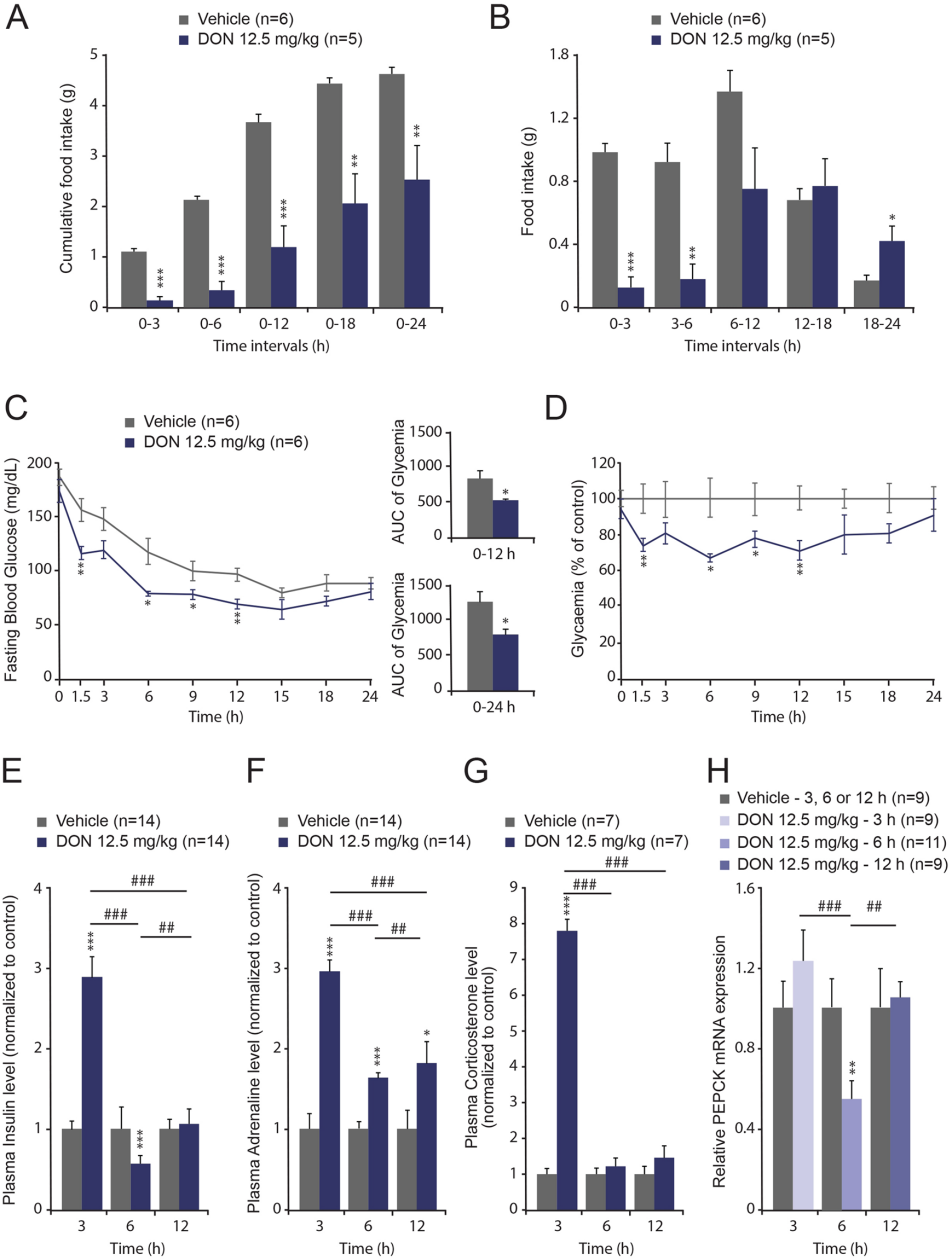
(**A**, **B**) Acute *p.o.* DON administration modifies food intake. Cumulative food intake (**A**) and food intake measured at different time intervals (**B**) over a 24 h period in mice *p.o.* administered either vehicle or DON (12.5 mg/kg bw). (**C**, **D**) DON-induced hypoglycemia. Time course of blood glucose concentration expressed in mg/dL (**C**) and normalized or DON (12.5 mg/kg bw, blue line, (**D**). Insets in (**C**) Area under the cure calculated for two time periods i.e. 0–12 h and 0–24 h. (**E**, **G**) Dosages of plasma insulin (**E**), adrenaline (**F**) and corticosterone (**G**) concentrations assayed for vehicle- and DON-treated animals. (**H**) mRNA expression of Pck1 assessed by qRT-PCR from the livers of control and DON-challenged animals for the indicated times. A two-way analysis of variance test was performed between time series (3, 6 and 12 h) of DON-treated and untreated mice (panels **A**, **B** and **E**–**H**). Comparisons between data from vehicle and DON-treated mice were performed using unpaired 2-tailed Student’s *t* test (panels **C**, **D**). *P < 0.05 **P < 0.01 and ***P < 0.001 significantly different from control mice. ^#^*P* < 0.05, ^##^*P* < 0.01 and ^###^*P* < 0.001 significant difference between time point of DON-treated mice.

Acute per os (p.o.) treatment with DON (12.5 mg/kg bw) induced a strong reduction of food intake in mice (Fig. 2A), as previously reported^10,13^. We observed that food consumption measured at different time intervals revealed an important reduction of food intake by DON, as early as the 0–3 h interval post-treatment. After 6 h, the DON-induced hypophagia regressed (Fig. 2B). We next analyzed the possible alteration in glycemic control under DON intoxication. Both DON and control groups were fasted during glycaemia measurements to exclude that DON-induced hypoglycemia resulted from differential food intake between the two experimental groups. As a consequence, both groups exhibited a continuous fall in glycaemia during the experiment (Fig. 2C). The anorexic dose of DON that we used induced a fall in blood glucose detected as soon as 1.5 h after *p.o*. DON administration and reached a maximum at 12 h post-treatment (Fig. 2C, D). Quantification of area under the curve (AUC) at 12 and 24 h confirmed the hypoglycemic action of DON (Fig. 2C). In parallel, we quantified plasma insulin (Fig. 2E), adrenaline (Fig. 2F) and corticosterone (Fig. 2G) levels at three time points after DON administration. In response to DON ingestion, plasma insulin, adrenaline and corticosterone concentrations increased as soon as 3 h post-treatment, but while insulin and corticosterone levels returned to basal level at 6 h (Fig. 2E, G), adrenaline remained significantly elevated during 12 h (Fig. 2F). Cytosolic phosphoenolpyruvate carboxykinase (PEPCK; encoded by *Pck1*) catalyzes the first committed step in gluconeogenesis. Classically, PEPCK gene expression is induced by catecholamines and glucocorticoids during periods of fasting, but is inhibited by insulin after feeding^22^. Here, Pck1 gene expression was reduced 6 h after DON treatment (Fig. 2H).

### DON intoxication and hepatic glycogen content

**Figure 3:**
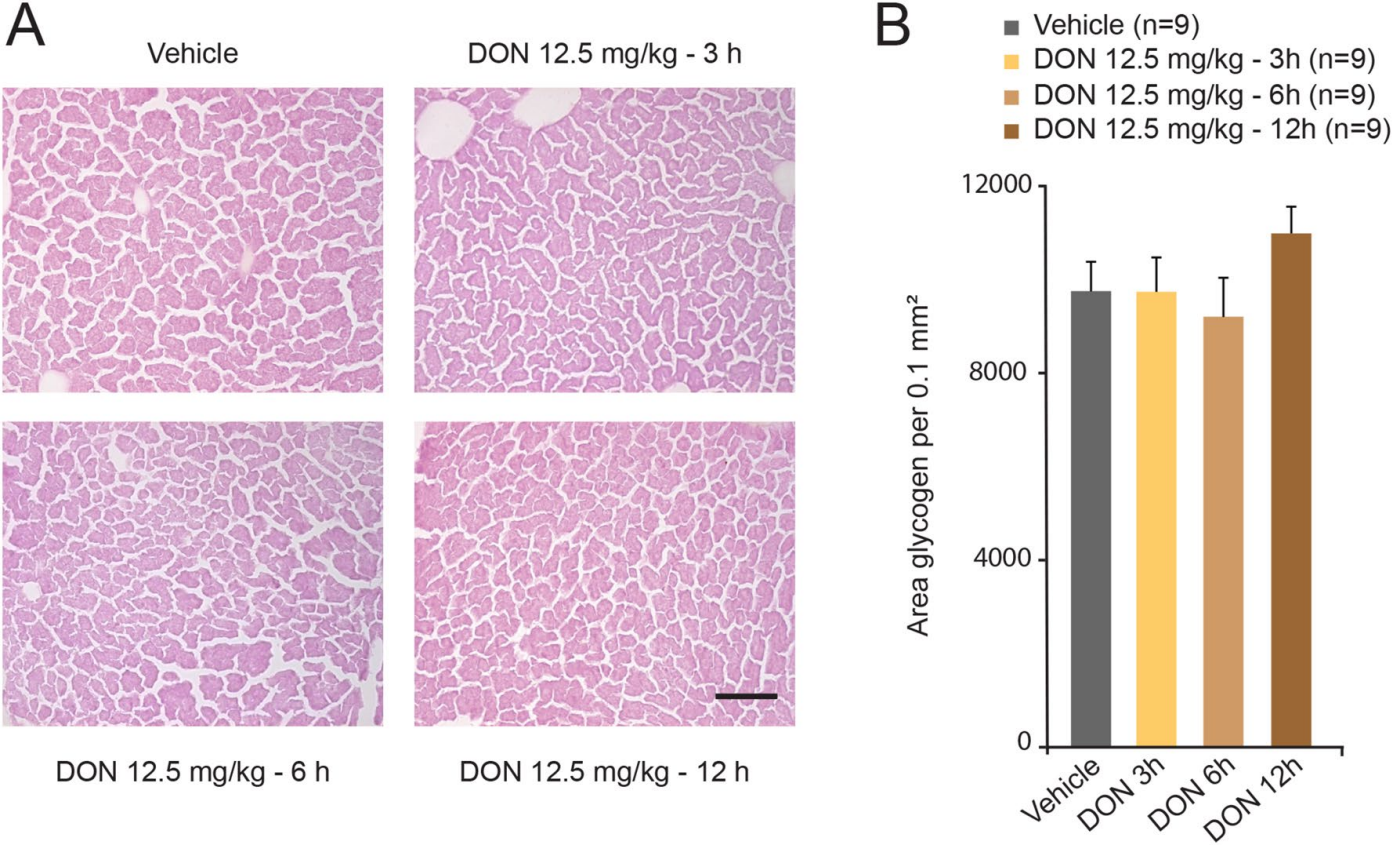
(**A**) Representative microphotographs of frozen liver section stained with PAS observed in vehicle (distilled water) or DON (12.5 mg/kg) treated animals for the indicated times. Scale bar = 100 μm. (**B**) Quantification of PAS positive staining per surface unit in liver of vehicle and DON-treated mice. A two-way analysis of variance test was performed between time series (3, 6 and 12 h) of DON-treated and untreated mice.

To determine whether loss of liver glycogen synthesis diverts glucose toward fat synthesis, liver tissues were stained with PAS. Stained hepatocytes were visible in liver tissues of all tested conditions (Fig. 3A). Quantification of staining intensity did not reveal a significant effect of DON whatever the time post-treatment considered (Fig. 3B).

### DON modulates activity of central autonomic centers

**Figure 4:**
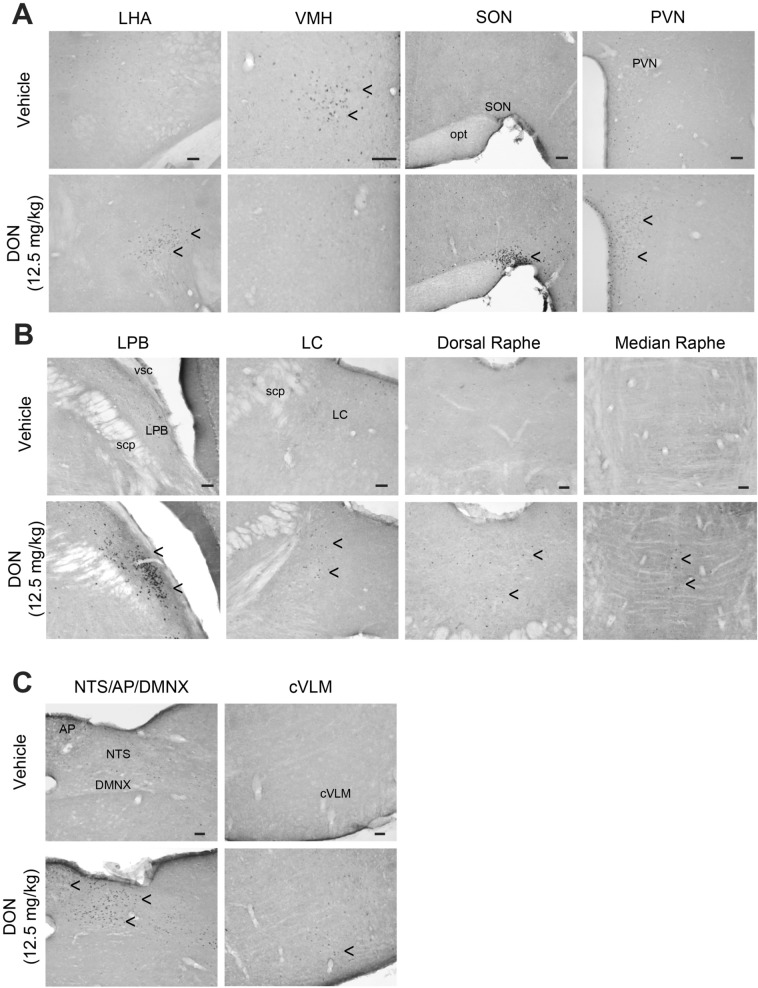
Representative coronal sections illustrating the c-Fos labeling observed within hypothalamic (**A**), pontic (**B**) and brainstem (**C**) regions of mice treated with water and DON (12.5 mg/kg *p.o.*). Animals were sacrificed 1.5 h after treatment. Arrowheads indicate areas of strong c-Fos labeling. AP, area postrema; cVLM, caudal ventrolateral medulla; DMNX, dorsal motor nucleus of the vagus; LC, locus coeruleus; LHA, lateral hypothalamic area; LPB, lateral parabrachial nucleus; opt, optic tract; PVN, paraventricular nucleus; NTS, nucleus tractus solitarius; SON, supra optic nucleus; scp, superior cerebral peduncle; VMH, ventromedial hypothalamus; vsc, ventral cerebrospinal tract. Scale bar: 100 µm.

**Figure 5:**
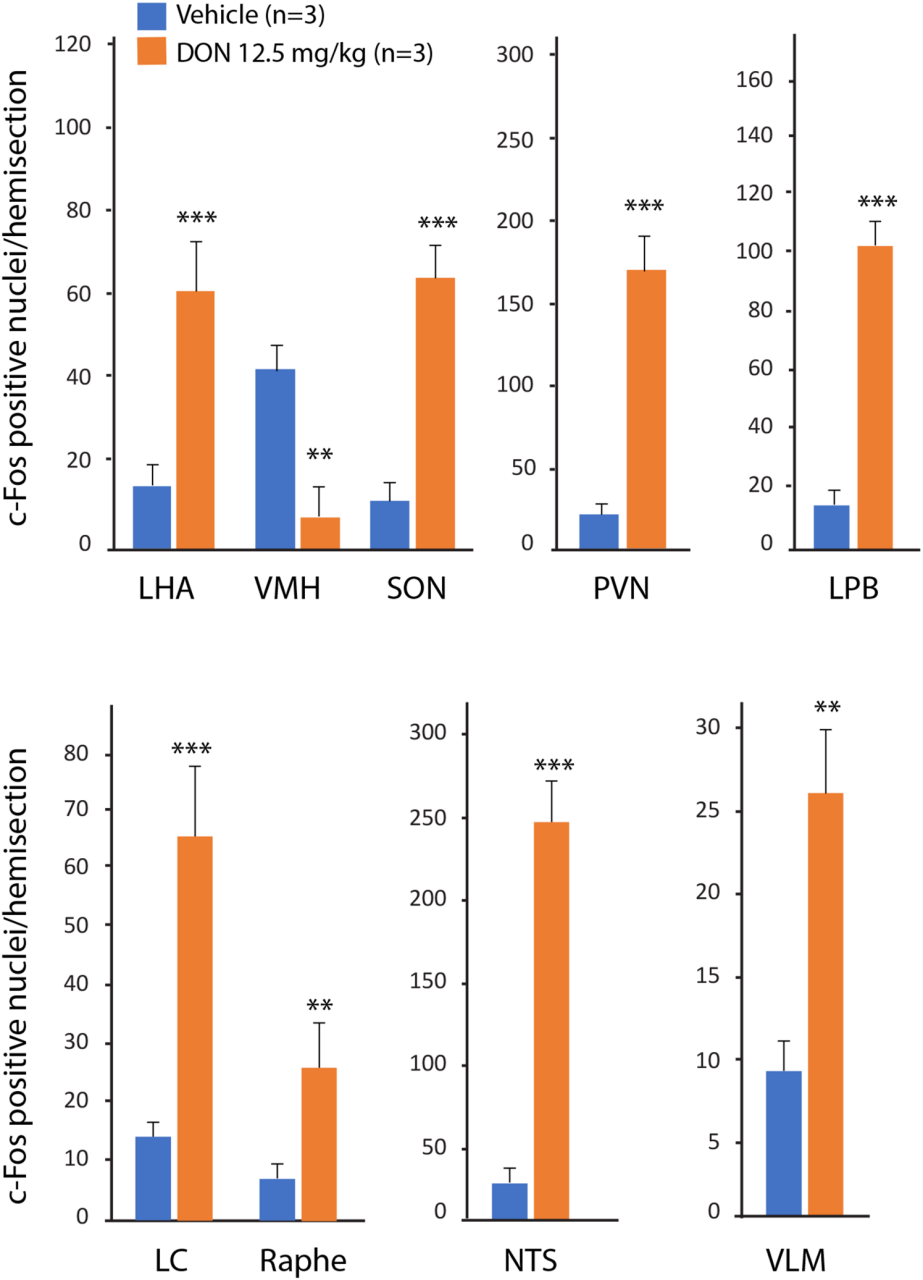
Quantification of c-Fos positive nuclei in central structures sensitive to DON treatment. LC, locus coeruleus; LHA, lateral hypothalamic area; LPB, lateral parabrachial nucleus; PVN, paraventricular nucleus; NTS, nucleus tractus solitarius; SON, supra optic nucleus; VLM, caudal ventrolateral medulla; VMH, ventromedial hypothalamus. Significances were determined relative to control animals. Comparisons between data from vehicle and DON-treated mice were performed using unpaired 2-tailed Student’s *t* test. **P < 0.01 and ***P < 0.001 significantly different from control mice.

Using immunohistochemistry, we detected the immediate early gene c-Fos as an indicator of central structures activated in response to DON treatment. A markedly low basal level of c-Fos-positive nuclei was observed in the brainstem and forebrain of water-treated mice with the noticeably exception of the VMH, a region associated to satiety, which exhibited a high level of c-Fos expression in control condition (Fig. 4A). Animals treated with DON (12.5 mg/kg *p.o.*) displayed c-Fos labeling in a limited number of structures (Figs. 4 and 5). A strong rise in c-Fos-positive cells was observed only in hypothalamic, pontine and brainstem nuclei (Fig. 4). Within the hypothalamus, the number of c-Fos-positive nuclei increased in the LHA (22.4 ± 7.5 versus 91.6 ± 17.7 positive nuclei for control and DON respectively), PVN (33.8 ± 10.6 versus 275.4 ± 34.8 positive nuclei for control and DON respectively) and was reduced within the VMH (61.5 ± 8.7 versus 11.2 ± 10.0 positive nuclei for control and DON respectively) when compared to control condition (Figs. 4A, 5). In pons area, raphe nuclei, LC and LBP exhibited also a rise in c-Fos positive cells in response to DON (Fig. 4B). Finally, at the brainstem level, the NTS, AP, DMNX and VLM were found to be strongly labeled 1.5 h post-treatment (Fig. 4B). In the NTS, the mean number of c-Fos positive nuclei per hemisection reached 227.1 ± 19.5 versus 21.8 ± 5.5 in control mice (Fig. 5).

### Hepatic expression of fatty acid oxidation genes is reduced during DON intoxication

**Figure 6:**
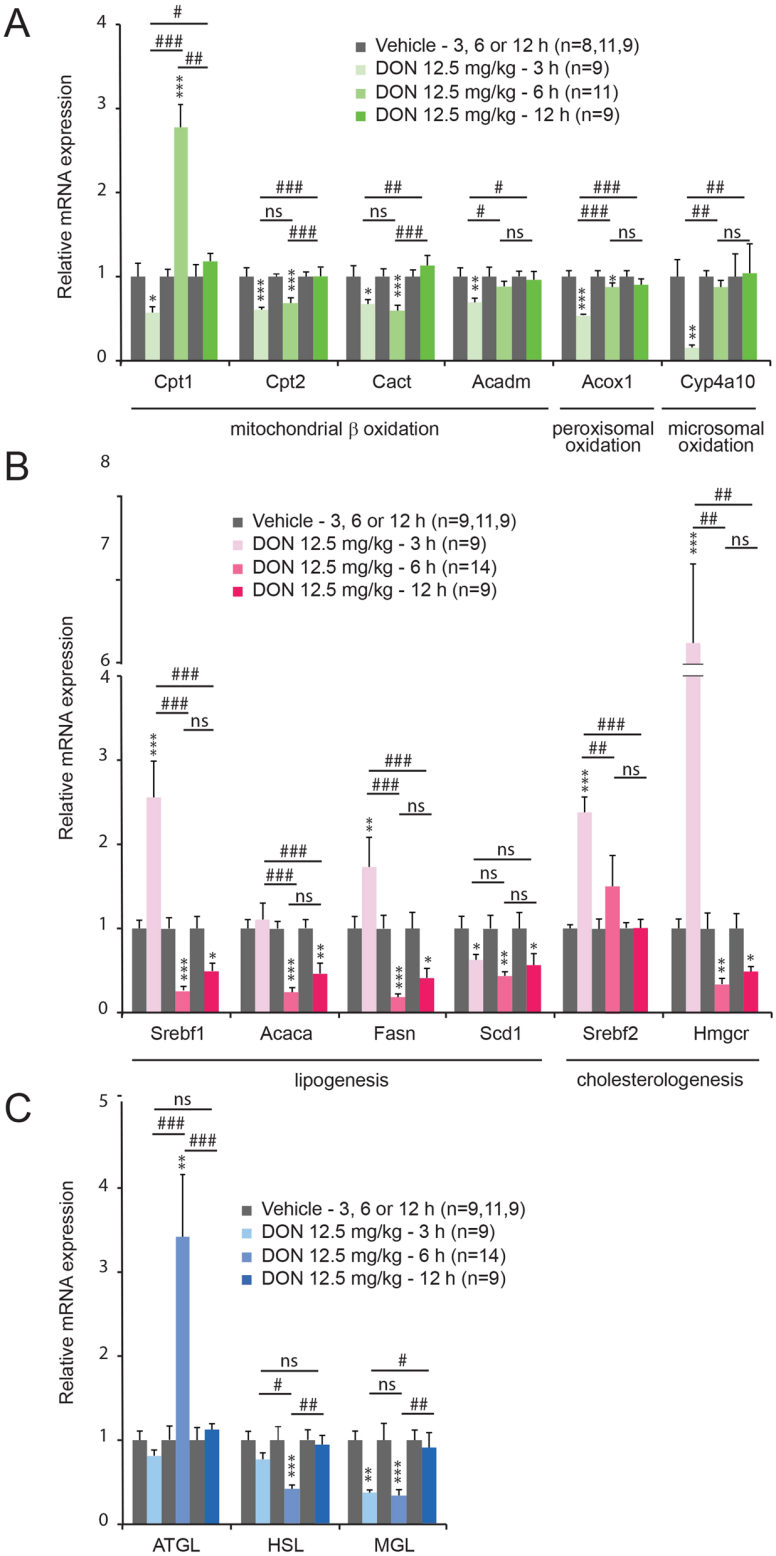
mRNA expression of the indicated FAox (**A**), lipogenic and cholesterologenic (**B**) and lipolytic (**C**) genes was assessed by qRT-PCR from the livers of control and DON-challenged animals for the indicated times. Significances were determined relative to control animals. A two-way analysis of variance test was performed between time series (3, 6 and 12 h) of DON-treated and untreated mice. *P < 0.05, **P < 0.01 and ***P < 0.001 significantly different from control mice. ^#^*P* < 0.05, ^##^*P* < 0.01 and ^###^*P* < 0.001 significant difference between time point of DON-treated mice. ns: no significant difference.

We used RT-qPCR to ask whether fatty acid oxidation (FAox) genes expression is modulated by DON. Genes encoding key steps in mitochondrial-oxidation (Cpt-1, Cpt-2, Cact, Acadm), peroxisomal oxidation (Acox1), and microsomal oxidation (Cyp4a10) were significantly reduced as early as 3 h after DON intoxication (Fig. 6A). Only Cpt-2 and Cact mRNA expression remained significantly reduced at 6 h after treatment. All genes returned to their basal level 12 h after treatment (Fig. 6A).

### Hepatic expression of lipogenic genes is altered during DON intoxication

We next examined, in control and DON-treated animals, the hepatic expression of genes encoding both transcriptional major regulators of lipogenesis and cholesterologenesis (Srebf1, Srebf2, respectively) and important and rate-limiting enzymes in both processes (Acaca, Fasn, Scd1, and Hmgcr). Srebf1 gene expression was increased after 3 h DON treatment. At this time point, mRNA coding rate-limiting enzymes involved in lipogenesis remained unchanged with the exception of Scd1 mRNA whose expression was reduced (Fig. 6B). Later i.e. 6 and 12 h after DON administration, Srebf1, Acaca, Fasn and Scd1 mRNA expression was strongly reduced. Regarding cholesterologenesis, Srebf2 and Hmgcr mRNA expression was robustly enhanced in 3 h DON-treated animals. At 6 and 12 h after DON administration, Srebf2 mRNA level returned to its basal level while Hmgcr mRNA expression was highly diminished.

### DON modulates differently expression of hepatic lipolytic genes

mRNAs coding ATGL which mainly hydrolyzes TG to release NEFAs and diacylglycerol (DAG) was strongly increased at 6 h after DON challenge but remained unchanged at 3 and 12 h (Fig. 6C). Conversely, HSL mRNA was reduced at 6 h post DON administration. HSL allows DAG hydrolysis to release NEFAs and monoacylglycerol (MAG). Moreover, at the time points 3 and 6 h after DON treatment, MGL mRNA expression was strongly reduced (Fig. 6C). MGL completely hydrolyzes MAG to produced glycerol and NEFAs.

### DON induced hepatic up-regulation of inflammation and ER stress related genes

**Figure 7:**
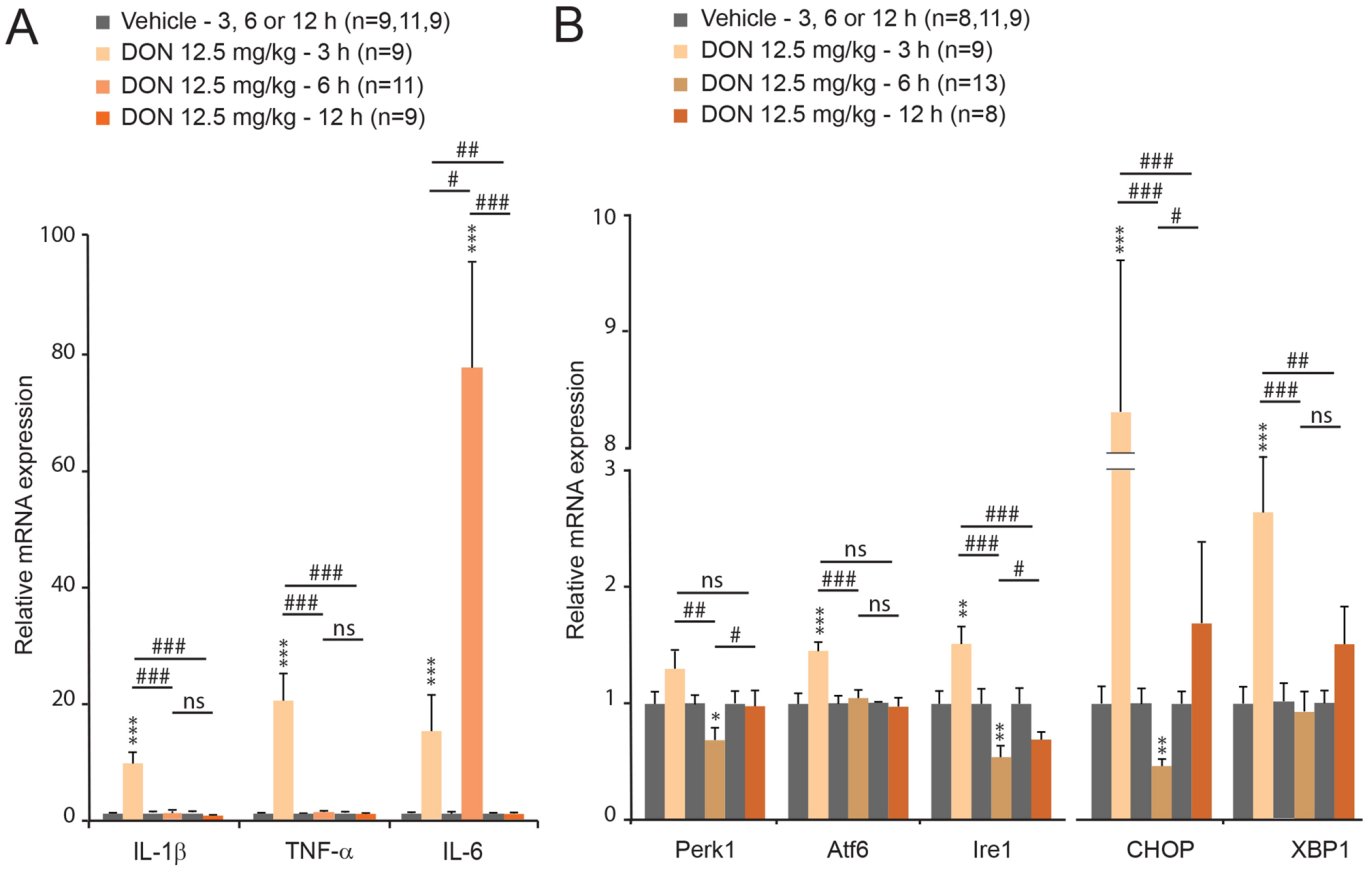
The expression of pro-inflammatory cytokines (**A**) and ER stress related genes (**B**) was measured by qRT-PCR from the livers of control and DON-challenged animals for the indicated times. A two-way analysis of variance test was performed between time series (3, 6 and 12 h) of DON-treated and untreated mice. * *P* < 0.05, **P < 0.01 and ***P < 0.001 significantly different from control mice. ^#^*P* < 0.05, ^##^*P* < 0.01 and ^###^*P* < 0.001 significant difference between time point of DON-treated mice. ns: no significant difference.

In addition to lipid metabolism linked genes, we analyzed expression of genes coding pro-inflammatory cytokines. A hallmark of DON toxicosis at moderate doses is the up-regulation of pro-inflammatory cytokines in various peripheral tissues^1,12^. As soon as 3 h after DON treatment, we observed a huge up-regulation of pro-inflammatory cytokines mRNA i.e. interleukine-1β (IL-1β), interleukine-6 (IL-6), tumor necrosis factor-α (TNF-α) in the liver (Fig. 7A). In the liver, ER stress leads to lipid accumulation, or hepatic steatosis^23–25^, indicating that ER stress and/or unfolded protein response (UPR) activation regulates, directly or indirectly, lipid metabolism in this organ. Accordingly, we quantified mRNA expression of three effectors proximal to the ER that sense and respond to the presence of unfolded and misfolded proteins i.e. ATF6, IRE1, PERK. DON treatment induced a small and transient up-regulation of ATF6 and IRE1 (Fig. 7B). As disruption of the ER is a powerful inducer of the transcription factors C/EBP homologous protein (CHOP) and full-length X-box-binding protein 1 (XBP1) mRNA, we analyzed CHOP and XPB1 mRNA expression in response to DON administration. CHOP and XBP1 mRNA expressions were transiently but strongly increased 3 h after DON (Fig. 7B).

### DON intoxication stimulates lipolysis related enzymes and pro-inflammatory cytokines genes expression in adipose tissues

**Figure 8:**
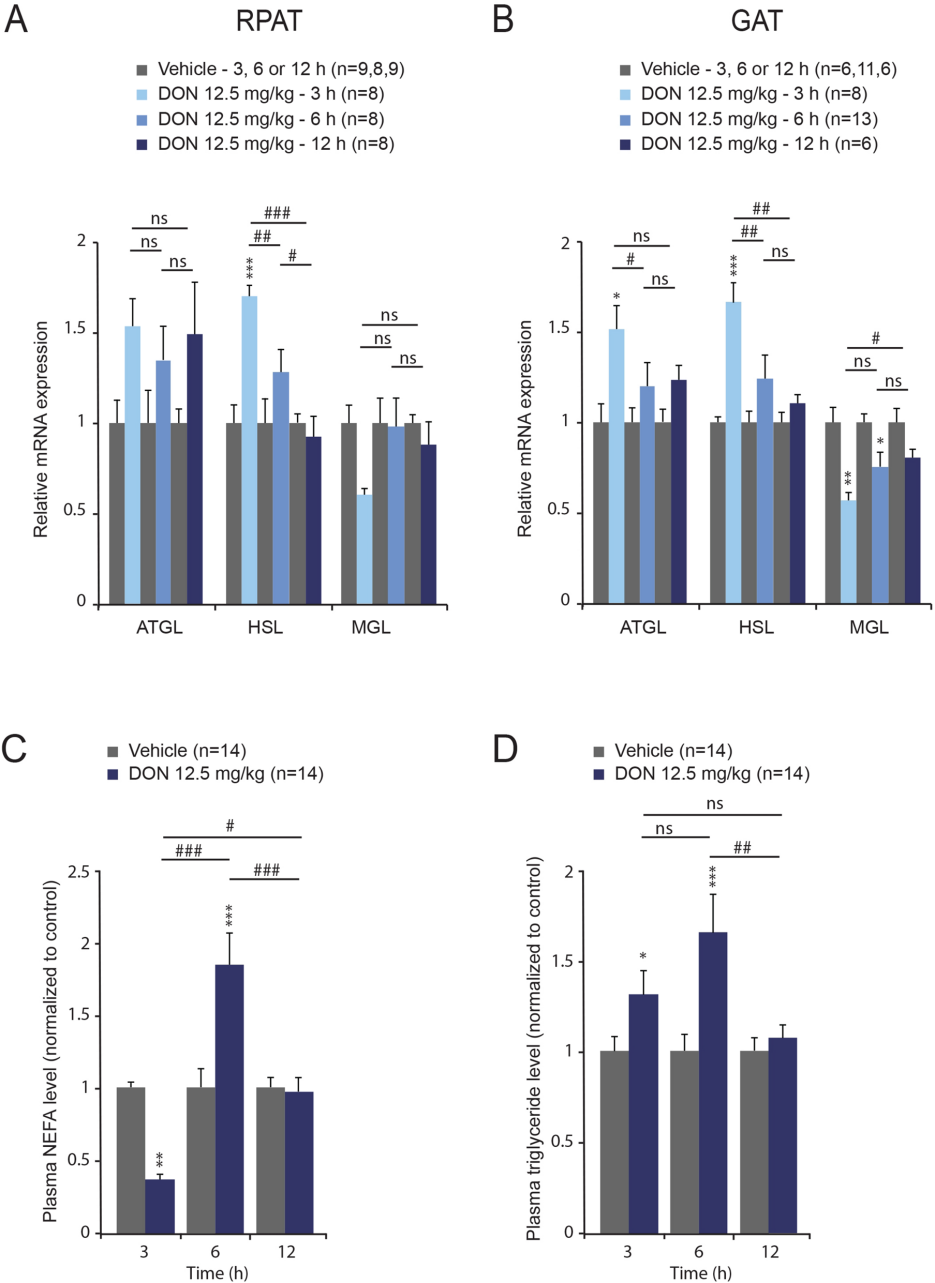
The expression of genes coding lipolytic enzymes was measured by qRT-PCR from RPAT (**A**) and GAT (**B**) of control and DON-challenged animals for the indicated times. Dosages of NEFAs (**C**) and TGs (**D**) concentrations assayed for vehicle- and DON-treated animals. A two-way analysis of variance test was performed between time series (3, 6 and 12 h) of DON-treated and untreated mice. *P < 0.05, **P < 0.01 and ***P < 0.001 significantly different from control mice. ^#^*P* < 0.05, ^##^*P* < 0.01 and ^###^*P* < 0.001 significant difference between time point of DON-treated mice. ns: no significant difference.

The increased lipid storage observed during DON intoxication led us to consider the possibility that this process is partly driven by lipolysis from adipose tissue. Thus, we analyzed mRNA expression of enzymes involved in lipolysis in GAT and RPAT tissues, respectively. In both adipose tissues, DON treatment induced an up-regulation of ATGL and HSL mRNA expression as early as 3 h (Fig. 8A, B). We quantified plasmatic NEFAs and TGs levels in response to DON administration. While NEFAs level was decreased after 3 h DON administration (Fig. 8C), both plasma NEFAs and TGs concentrations increased at 6 h (Fig. 8C, D).

**Figure 9:**
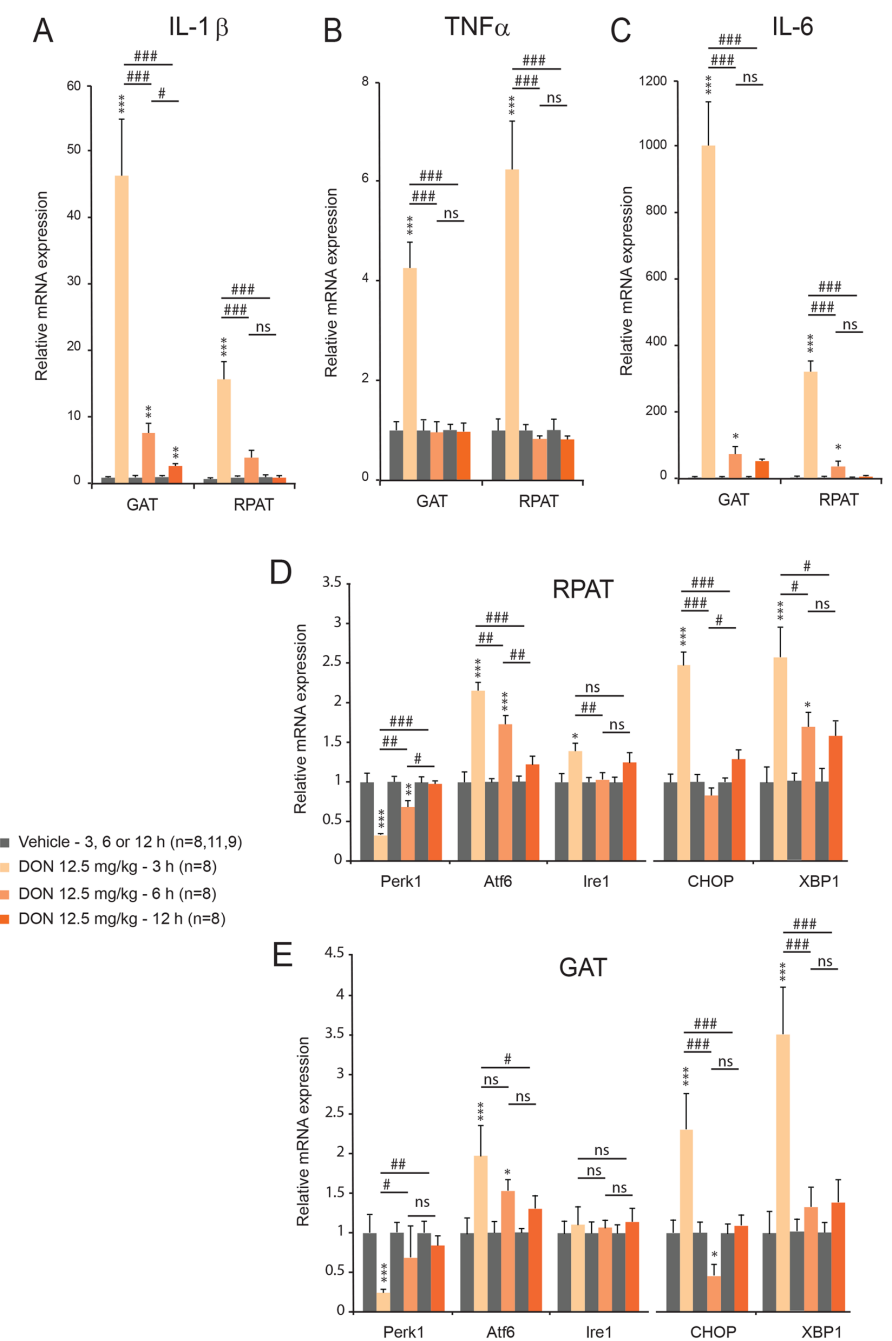
The expression of IL-1α, TNF-β (**B**) and IL-6 (**C**) mRNA and ER stress related genes (**D**, **E**) was measured by qRT-PCR from RPAT and GAT of control and DON-challenged animals for the indicated times. A two-way analysis of variance test was performed between time series (3, 6 and 12 h) of DON-treated and untreated mice. *P < 0.05, **P < 0.01 and ***P < 0.001 significantly different from control mice. ^#^*P* < 0.05, ^##^*P* < 0.01 and ^###^*P* < 0.001 significant difference between time point of DON-treated mice. ns: no significant difference.

Moreover, we observed that DON administration induced a robust increase in IL-1β, TNF-β and IL-6 mRNA expression within both GAT and RPAT. Each cytokines mRNA expression peaked at 3 h and progressively returned to their basal level (Fig. 9A–C). As performed at the liver level, we quantified mRNA expression of three effectors of the UPR response i.e. ATF6, IRE1, PERK. DON treatment induced a small and transient up-regulation of ATF6 (Fig. 9D). We next analyzed CHOP and XPB1 mRNA expression in response to DON administration. CHOP and XBP1 mRNA expressions were transiently but strongly increased 3 h after DON (Fig. 9D, E).

## Discussion

Although DON was previously reported to compromise regulation of energy metabolism^8,11^, the underlying mechanisms are still poorly understood. Moreover, it is largely admitted now that a significant percentage of the human population is chronically exposed to DON doses which can exceed the provisional maximum tolerable daily dose particularly in infants and children^3–7^. This argues for further studies investigating DON effects on the regulation of energy balance. In the present work, we observed that an anorexigenic dose of DON (12.5 mg/kg bw) induced a rapid (i.e. significant after 6 h) lipids accumulation in the liver, as attested by ORO inclusion coloration, and ADRP expression increase. ADRP surrounds the lipid droplets, binds phospholipids with high affinity^26^ and is involved in assisting the storage of neutral lipids within the lipid droplets^27^. Interestingly, increased liver steatosis was associated with hypoglycemia and increased plasma insulin concentration. Such situation appears unusual and may reflect a global metabolic imbalance. Classically, in a fasted state, plasma insulin concentration falls and fuel substrates (glucose and TG) are released from the liver into blood circulation. NEFAs released from adipose tissues through lipolysis are also oxidized in hepatic mitochondria through fatty acid β-oxidation to provide energy to hepatocytes and also ketone bodies which are exported into the circulation. So, liver glucose and ketone bodies supply essential metabolic fuels for extrahepatic tissues during starvation. The increased insulin release observed here during DON intoxication reflect a deep disturbance of the autonomic nervous system functioning. Supporting this hypothesis, using c-Fos expression as cellular activation marker^28^, we noticed a reduced number of activated neurons within the VMH of DON-treated animals. Interestingly, insulin secretion by the β cell of the endocrine pancreas is under an inhibitory tonus by the sympathetic nervous system. More precisely, bilateral VMH lesions induced activation of the parasympathetic outflow and hyperinsulinemia^29^. Reduced VMH c-Fos expression in response to DON could explain the early hyperinsulinemia and consecutive hypoglycemia. This increased insulinemia in response to DON could alternatively reflect a direct stimulation of pancreas secretion by the toxin. The two hypotheses are not mutually exclusive. To date, very few clues on the possible direct action of DON on pancreas exist^30^ but this possibility deserves to be tested in the next future. In turn, this DON-induced hyperinsulinemia may contribute to liver steatosis since insulin is known to stimulate hepatic lipogenesis and to reduce β-oxidation^31^. We also observed a strong activation of other prototypic neuronal nuclei of the sympathetic autonomic nervous system and hypothalamic–pituitary–adrenal axis including LHA, PVN, raphe nuclei and brainstem centers which could explain the high plasmatic adrenaline and corticosterone levels. Alternatively, visceral pain suspected to might contribute to adrenal gland activation and contribute to the rise in plasmatic adrenaline and corticosterone. Consecutively, adrenaline and corticosterone may influence liver and adipose tissue metabolism. The DON-induced hypoglycemia may also participate in the activation of sympathetic outflow^32^. It should be noted that despite this increase in plasma adrenaline and corticosterone levels, DON-treated animals failed to regulate correctly their glycaemia suggesting a possible alteration of hepatic glucose synthesis. Hepatic glycogen stores have long been known to decrease with starvation^32^. No reduction in glycogen content during DON treatment was observed here. This was in accordance with DON-induced hypoglycemia and VMH inhibition since VMH-liver sympathetic pathway was reported to cause glucose output from the liver which results in hyperglycemia and a marked reduction of hepatic glycogen^33^.

The hepatic accumulation of lipids raised the question about their origin. A modification of hepatic lipogenesis and lipolysis could explain DON induced steatosis (see below). Another source for hepatic free fatty acids is lipids recruitment from the plasma lipids pool. Indeed, NEFAs released from adipose tissue through lipolysis are the main source for liver TG pools^34^ and the only major site of NEFAs liberation into plasma is adipose tissue. Moreover, in the fasting state, plasmatic NEFAs arise almost entirely from TG hydrolysis within the adipocyte^35^. The increase in NEFAs and TG plasmatic levels detected in response to DON supports the hypothesis that lipid mobilization from adipose tissue likely drives hepatic steatosis. Adrenaline and glucocorticoids are well-known to stimulate lipolysis in adipose tissue during fasting or exercise, while insulin represses it^36,37^. The increase in adrenaline and corticosterone release observed here during DON intoxication could mobilize lipids from adipose tissues and in turn partly drive hepatic steatosis.

### Transcriptional alterations of lipid and glucose metabolic pathways by DON

DON is reported to bind to the peptidyl transferase region of the 28S ribosomal RNA resulting in ribotoxic stress^38^. As a result, many pathways that play a role in ribosomal stress such as ribosomal RNA biosynthesis, ribonucleoprotein complex, and protein synthesis are up-regulated by DON^39,40^. Recently transcriptional studies have also reported that DON modulated genes expression in a variety of models such as Jurkat cells, peripheral blood mononuclear cells^39^, chondrocytes^41^, murine macrophage^42^, intestinal epithelial cells^43^ or *Caenorhabditis elegans*^44^. A number of genes are significantly affected by DON after 3 and 6 h of exposure, affecting a wide variety of processes and pathways^40^. In order to decipher the mechanisms leading to hepatic steatosis in response to DON, we analyzed the expression of genes coding key enzymes regulating lipid and glucose metabolism. Indeed, the excessive fat accumulation in the liver could originate from an altered balance between anabolic and catabolic lipid pathways and/or from an increased lipolysis of peripheral fats stored in white adipose tissue that flow to the liver as NEFAs^45^. To test the first hypothesis, we first analyzed genes encoding key steps in mitochondrial (Cpt-1, Cpt-2, CACT, Acadm), peroxisomal (Acox1), and microsomal oxidation (Cyp4a10). All these hepatic genes expression was diminished by DON exposure as soon as 3 h, suggesting a reduced lipid oxidation capability in the liver of DON treated animals. This reduced lipids consumption could in turn contribute to hepatic steatosis development. Regarding lipogenesis (Srefb1, Aacaca, Fasn and Scf1) and cholesterologenesis (Srebf2 and Hmgcr) linked genes expression, a biphasic response was observed with an initial up-regulation, particularly for genes involved in cholesterologenesis, followed by a down-regulation. This revealed a transient stimulation of hepatic cholesterol and lipids synthesis, accelerating lipids accumulation immediately after DON exposure. A more contrasting picture is given for lipolysis-linked genes expression. Lipolysis is defined as the sequential hydrolysis of triacylglycerol catalyzed by three successive enzymes. ATGL catalyzes the initial step in TGs lipolysis to produced DAG and one molecule of fatty acid. Working in concert with ATGL, HSL converts DAG to MAG and NEFAs, and MGL hydrolyzed MAG into glycerol and NEFAs, respectively^46^. In addition to its role in adipose tissue TG mobilization, ATGL plays crucial roles in regulating lipid homeostasis in other tissues including liver. While expressed at low levels in the liver, ATGL is physiologically relevant for efficient hepatic lipid metabolism, since loss of hepatic ATGL causes hepatic steatosis^47^. ATGL coding gene (Pnpla2) expression is up-regulated 6 h after DON administration but remained unchanged at 3 and 12 h most likely to struggle the excessive lipids accumulation which reached a maximum at 6 h post treatment. But surprisingly, HSL and MGL mRNAs were rather repressed during DON exposure suggesting a more complex scheme in hepatic lipolytic pathways. PEPCK which catalyzes the first committed step in gluconeogenesis is down-regulated by DON treatment (6 h) suggesting an altered gluconeogenesis that could partly explain DON-induced hepatic lipids accumulation and long-lasting hypoglycemia^48^.

Moreover, within the adipose tissues, ATGL and HSL mRNAs were significantly up regulated at 3 h after DON administration, while MGL mRNA expression was reduced. In periods of fasting or high energy demands, the hormonal stimulation of adipocytes, mainly by catecholamines, leads to a series of intracellular reactions that result in the activation of ATGL and HSL enzymes^46^. The up-regulation of both ATGL and HSL mRNAs observed here could reflect the mobilization of these pathways and illustrate an increased lipolytic activity within the adipose tissues. Interestingly, together, ATGL and HSL are responsible for about 95% of the hydrolysis of TG and HSL may revert partially a reduced MGL activity^49^. Furthermore, as mentioned above, the increased concentration of plasma NEFAs at 6 h after DON treatment seems to confirm the contribution of adipose tissues lipolysis in the development of liver steatosis.

### DON intoxication and NAFLD: possible links?

In addition to hepatic accumulation of fatty acid, we observed that DON induced a strong up-regulation of cytokines mRNA i.e. IL-1β, TNF-α and IL-6 within the liver and adipose tissues. These results were consistent with the previously reported pro-inflammatory action at low DON doses^11,41,42^. This increased cytokines production within the adipose tissues could contribute to ectopic lipid deposits since pro-inflammatory cytokines were reported to stimulate adipocyte lipolysis and fatty acids release^50^. Moreover, it was proposed that DON by acting as a ribotoxin could increase the quantity of unfolded proteins thereby evoking an ER stress. ER stress and UPR could in turn stimulate pro-inflammatory cytokines production^8^. UPR operates through three effector proteins proximal to the ER, that sense and respond to the presence of unfolded and misfolded proteins: ATF6, IRE1, and PERK. Downstream of these effectors, CHOP and XBP1 act as critical mediators connecting accumulation and aggregation of unfolded proteins in the ER and oxidative stress to apoptosis^51,52^. Interestingly, we observed a transient upregulation of ATF6, IRE1, CHOP and XBP1 mRNAs in the first hours following DON intoxication within both the liver and adipose tissues, suggesting that DON induces an ER stress in these tissues. Consistently, a previous study has reported that DON induces ribotoxic stress, ER stress and deficient UPR^53^. It should be also noted that CHOP over expression could be induced by glucose deprivation since glucose deprivation induces ER stress by inhibiting N-linked protein glycosylation^54^. Thus, the induction of ER stress/UPR could be here secondary to the chronic DON-induced hypoglycemia. Previous reports using cultured hepatocytes have suggested that ER stress stimulates lipogenesis^55^. Moreover, XBP1 was also reported to drive the transformation from glucose to lipid^56^. It is thus possible that DON-induced ER stress may account for the transient up-regulation of lipogenesis and cholesterologenesis linked-genes and for a stimulation of lipogenesis only during a transient window corresponding to the first hours after intoxication. Finally, it can be also envisaged that DON-induced hepatic accumulation of lipids can subsequently leads to cellular stress and UPR since chronic lipid accumulation in the liver has been previously shown to stimulate ER stress, and subsequently to trigger the UPR^57^.

The concomitant induction by DON of ER stress, inflammation and excessive hepatic lipids accumulation is evocative of a non-alcoholic fatty liver disease (NAFLD). A condition of simple fatty liver, refereed as non-alcoholic fatty liver (NAFL), is a benign condition characterized by hepatic lipid accumulation and commonly associated with obesity. However, NAFL can progress to non-alcoholic steatohepatitis (NASH) which can lead to hepatocyte injury (necrosis, fibrosis and inflammation) and cirrhosis, resulting in increased morbidity and mortality. The entire spectrum of these hepatic lesions is referred to as non-alcoholic fatty liver disease (NAFLD). The transition of simple fatty liver to NASH seems to be favored by several genetic, environmental factors and drugs^58–60^. Long-lasting exposure to fatty acids and their deposits, insulin resistance and chronic low-grade inflammation seem to drive NASH progression during obesity^61^. Investigations into the molecular etiology of NASH have revealed that several putative signaling mechanisms, including the accumulation of reactive oxygen species (ROS), ER stress, and increased inflammation, can explain the lipotoxicity and progressive liver damage^58–60^. Altogether, these data question about the possible contribution of DON in the transition of NAFL to NASH, particularly in some vulnerable patients, as reported for those under some drugs treatments including halothane, methotrexate, rosiglitazone or tamoxifen^62^. Thus, there is an urgent need to evaluate the risk that DON repeated consumptions could induce more severe and/or more frequent acute liver injury in obesity models and worsen the transition of simple fatty liver to NASH.

**Figure 10:**
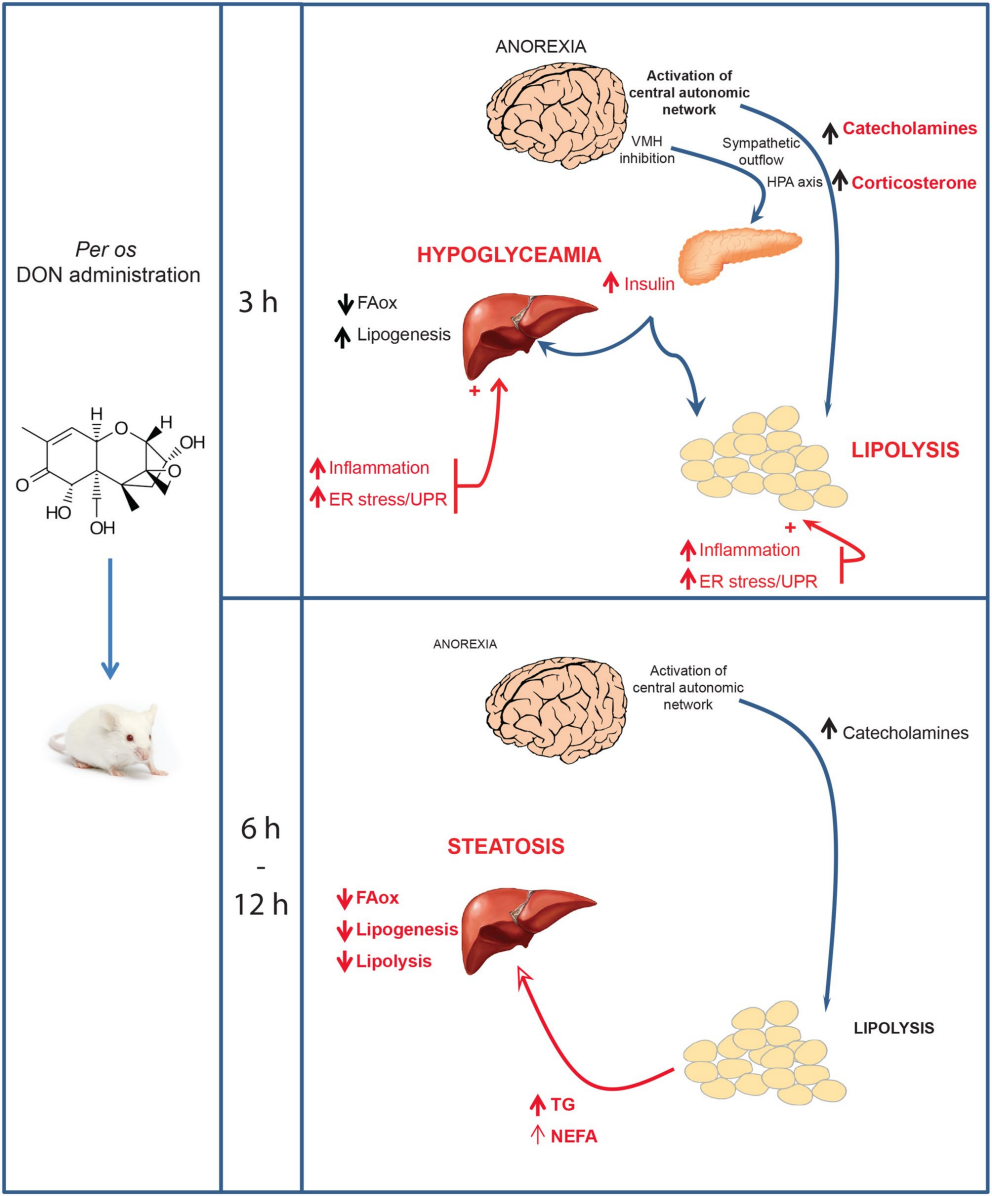
Schematic representation of the signaling events that might underlie the toxic effects elicited by DON intoxication on energy metabolism and liver steatosis. In an initial phase (3 h), DON induced a hormonal storm (insulin, adrenaline and corticosterone release) associated with a rise in liver lipogenesis and adipose tissue lipolysis. Moreover, inflammation and ER stress were observed within the liver and adipose tissues. A second phase intervened between 6 and 12 h where the increased blood NEFAs and TG resulting from adipose lipolysis and the drop in liver beta oxidation and lipolysis drive liver steatosis.

In summary, our data revealed that the food contaminant DON strongly disturbs hormonal, neuronal and metabolic functions resulting in an atypical state where anorexic, hyperinsulinemic and hypoglycemic DON-treated animals displayed an excessive accumulation of lipids within the liver (Fig. 10). Our results suggest that DON action can be divided into two sequential steps (1) an initial phase occurring within the first 3 h after administration where DON induced an increased insulin, adrenaline and corticosterone release, a rise in liver lipogenesis and adipose tissue lipolysis associated with inflammation and ER stress; and (2) a second subsequent phase (6–12 h) where despite normalization of insulinemia and inflammation, the increased blood NEFAs and TG resulting from adipose lipolysis drives liver steatosis, a steatosis amplified by the drop in liver beta oxidation and lipolysis. All these events can trigger mechanisms leading to NAFLD. Hence, we believe that further studies are required to evaluate the liver toxicity level of chronic exposure to this toxin especially in vulnerable individuals such as obese and diabetics.

## Abbreviations

Acaca: Acetyl-CoA carboxylase Alpha
Acadm: Acyl-CoA Dehydrogenase Medium Chain
ADRP: Adipose Differentiation-Related Protein
AP: Area postrema
ATF6: Activating transcription factor 6
ATGL: Adipose triglyceride lipase
CACT: Carnitine/Acylcarnitine Translocase (SLC25A20)
CHOP: C/EBP homologous protein
Cpt-1: Carnitine Palmitoyltransferase 1
Cpt-2: Carnitine Palmitoyltransferase 2
DMNX: Dorsal motor nucleus of the vagus
Fasn: Fatty acid synthase
GAT: Gonadic adipose tissue
Hmgcr: 3-Hydroxy-3-Methylglutaryl-CoA Reductase
HSL: Hormone-sensitive lipase
IL-1β: Interleukine-1β
IL-6: Interleukine-6
IRE1: Inositol-requiring enzyme 1 alpha
LBP: Lateral parabrachial nucleus
LC: Locus coeruleus
LHA: Lateral hypothalamic area
MGL: Monoacylglycerol lipase
NTS: Nucleus tractus solitarius
PCK1: Phosphoenolpyruvate Carboxykinase 1
PERK: Protein kinase R-like ER protein kinase
RPAT: Retroperitoneal adipose tissue
Scd1: Stearoyl-CoA Desaturase
Srebf1: Sterol Regulatory Element Binding Transcription Factor 1
Srebf2: Sterol Regulatory Element Binding Transcription Factor 2
TNF-α: Tumor necrosis factor-α
VLM: Caudal ventrolateral medulla
VMH: Ventromedial hypothalamus
